# Differential Role of Hypoxia-Inducible Factor 1 Alpha in Toll-Like Receptor-Mediated and Allergic Inflammatory Reactions

**DOI:** 10.1097/WOX.0b013e3181f8daa5

**Published:** 2010-10-15

**Authors:** Vadim V Sumbayev, Sally A Nicholas, Bernhard F Gibbs

**Affiliations:** 1 University of Kent, Anson Building, Central Avenue, Chatham Maritime, Kent, UK

**Keywords:** inflammation, allergy, Toll-like receptors, basophils, hypoxia, HIF-1

## Abstract

Hypoxia-inducible factor 1 (HIF-1) is a transcription complex that plays a pivotal role in cellular adaptation to hypoxic conditions. The role of this factor in inflammatory reactions associated with infections and allergies has recently become evident. In this review we summarize our current knowledge concerning the accumulation and role of HIF-1 in Toll-like receptor-mediated and allergic inflammation. The differential molecular mechanisms used to stabilize this protein in various settings and its ability to support both proinflammatory and angiogenic responses suggest important functional roles in both innate immune responses and allergies. Importantly, the HIF-1 transcription complex is activated in human basophils during IgE-mediated inflammatory responses. It is involved in VEGF expression and subsequent promotion of angiogenesis and in controlling energy metabolism.

## Introduction

Normal and pathologic functioning of the human immune system is always associated with the inflammatory process. During inflammation immune cells concentrate in tissue sites affected by injury with a pathogen or allergen [1,2]. Such processes give rise to an increased blood supply to the injured area followed by an increase in capillary permeability and leukocyte migration into the tissue [1,2]. These events therefore lead to decreased oxygen availability in these inflamed tissues where the effector cells are exposed to a hypoxic environment. In addition, during inflammatory reactions the intracellular oxygen consumption is increased and this is associated with an increase in the activity of ATP-degrading kinase cascades. Therefore, hypoxia becomes the normal physiological environment of inflammatory processes,[3,4] which includes several stages [5]:

1. Interaction of proinflammatory ligands with plasma membrane-associated or endosomal receptors such as Toll-like receptors (TLRs) that cause intracellular inflammatory stress.

2. Adaptation of the inflammatory effector cells to stress associated with low oxygen availability.

3. Ligand-induced inflammatory receptor-mediated expression of proinflammatory cytokines, which promote further stages of host immune defense or allergic reactions.

The key stage of the inflammatory process is adaptation of the effector cells to inflammatory stress. Nonadapted cells will be quickly eliminated by the activation of cell death mechanisms [5]. Therefore, upon interaction with proinflammatory ligands, the effector cells use adaptation mechanisms that allow them to survive and act under hypoxic conditions. This report summarizes our current knowledge about the role of hypoxic signaling pathways driven by the hypoxia-inducible factor 1 (HIF-1) transcription complex [3] in adapting effector cells to inflammatory stresses caused by infectious and allergic inflammation.

## Signaling receptors regulating infectious and allergic inflammation

TLRs are key pattern-recognition receptors (PRRs) that allow immune cells to specifically detect pathogen-associated molecular patterns and induce inflammatory responses to them [6-8]. Because of the ability of TLRs to specifically recognize pathogen-associated ligands, they lie at the core of host resistance to infectious disease. Examples of TLR ligands include lipopolysaccharides (LPS; recognized by TLR4), lipopeptides and peptidoglycan (PGN, recognized by cooperation of TLR2 with TLR1 or TLR6), flagellin (specific TLR5 ligand), viral single- or double-stranded RNA (detected by TLR7/TLR8 and TLR3, respectively), and bacterial or viral DNA containing CpG motifs (TLR9 ligands) [1,7-9]. TLR activation results in the recruitment of signaling intermediates, which include myeloid differentiation factor-88 (MyD88), Toll-interleukin (IL)-1 receptor (TIR)-associated protein (TIRAP, also known as MAL), Toll receptor-associated activator of interferon (TRIF), Toll receptor-associated molecule (TRAM), IL-1 receptor-associated kinases (IRAK), and tumor necrosis factor (TNF) receptor-associated factor 6 (TRAF6) [6,8]. TLRs are expressed in many leukocytes and tissue cell types, while the key effectors of inflammation/innate immunity are myeloid macrophages, neutrophils, and dendritic cells [6,8,10].

However, pathogens are not the only cause of inflammatory reactions. A number of human disorders such as allergic asthma, atopic dermatitis, allergic rhinitis, heyfever, and many others are associated with nonpathogenic inflammatory reactions [2]. This type of inflammatory response is known as allergic inflammation, the key effectors of which are basophils and mast cells [2,11]. Both types of cells are granulated and play different roles in adaptive and innate immune responses including promotion and regulation. Mast cells were found to express different angiogenic factors during inflammation and as such stimulate the process of angiogenesis [2,12,13]. Currently, it was reported that basophils play a critical role in inflammatory angiogenesis, mostly via the expression of several isoforms of vascular endothelium growth factor (VEGF) and its receptors [2,14-18]. Basophils and mast cells are 2 distinct hematopoietic lineages that play complementary or overlapping roles during acute and chronic immunoglobulin E (IgE)-associated allergic inflammatory reactions [2,14].

Both basophils and mast cells express the tetrameric form of the plasma membrane-associated high-affinity receptor Fc*ε*RI. This receptor recognizes IgE, inducing downstream signaling events leading to the allergic inflammatory response. Both basophils and mast cells are crucial effectors of T-helper 2 (T_H_2) cell-dependent, IgE-associated hypersensitivity reactions. Activated human basophils and mast cells express and release T_H_2 cytokines such as IL-13 and IL-4 that polarize immune responses, and produce biochemical mediators such as histamine and lipid metabolites (eg, leukotrienes), which are crucial for both regulation of immune reactions and chemotaxis [2,11,13,14,16,19].

It is important to mention that basophils also express TLRs, especially TLR2, 4, 9 and 10 and respond to some of the TLR ligands [20]. Therefore, it is likely that basophils recruit stress adaptation signaling pathways similar to those observed in the innate immune cells. As such, similar molecular mechanisms might participate in both infectious and allergic inflammation. Intriguingly, recent evidence clearly demonstrated that in both kinds of inflammation, the innate immune cells and basophils/mast cells recruit HIF-1 transcription complex during their adaptation to inflammatory stress.

## HIF-1 transcription complex

HIF-1 is a transcription complex consisting of 2 subunits, inducible HIF-1*α *and constitutive HIF-1*β *this subunit as also acts as aryl hydrocarbon receptor nuclear translocator or ARNT) [21]. Under normal oxygen conditions, HIF-1*α *protein undergoes regulated ubiquitination that causes its proteasomal degradation. HIF-1*α *interacts with the von Hippel-Lindau protein (pVHL), interacting with its oxygen-dependent degradation domain and targeting the protein for proteasomal degradation. VHL is also associated with elongins B and C, cullin-2, and possibly other factors that belong to E3 ubiquitin ligase functioning as a multiprotein complex. Interaction between pVHL with HIF-1*α *protein is regulated via hydroxylation of 402/564 proline residues. This process is catalyzed by HIF-1*α *prolyl hydroxylase (PHDs) enzymes [21]. HIF-1*α *PHDs recruit molecular oxygen, iron (II), 2-oxoglutarate (2-OG), and ascorbic acid during their catalytic action. Under hypoxic conditions prolyl hydroxylation of the HIF-1*α *subunit is down-regulated, and therefore the protein undergoes rapid stabilization [21].

There is now increasing evidence regarding the accumulation of HIF-1*α *protein followed by HIF-1 transactivation in different types of innate immune cells in response to TLR-ligand interactions [4,22-25]. Intriguingly, both membrane-associated and endosomal TLRs were found to trigger activation of the HIF-1 transcription complex [5]. Furthermore, Fc*ε*RI was also reported to use HIF-1 in its downstream signaling [18]. However, differential biochemical mechanisms are used in these cases to induce HIF-1*α *accumulation and HIF-1 transactivation.

## Molecular mechanisms of HIF-1 activation in human immune cells during infectious and allergic inflammation

The molecular mechanisms leading to the HIF-1*α *accumulation have been studied mostly for TLR4 (cell membrane associated) and TLRs 7 and 8 (endosomal receptors). In the case of TLR4 a redox-dependent mechanism is one of the contributors to the accumulation process [26-28]. In this case direct phosphorylation of the protein is also required for successful accumulation/transactivation of the HIF-1*α *protein [26]. Furthermore, an increase in HIF-1*α *mRNA was also detected [22,26]. After interaction with LPS, the intracellular TIR domain of the TLR4 binds myeloid differentiation factor 88 (MyD88), which recruits Bruton disease (Btk). Before the interaction with MyD88, Btk undergoes phosphorylation by Src and most likely other tyrosine kinases. Upon interaction with MyD88, Btk phosphorylates TIRAP, which interacts with MyD88 with high affinity [29,30]. Being a part of the multiprotein complex described above, Btk phosphorylates PI-3-specific phospholipase C (PLC-1γ), which then gains catalytic activity [26,29,30] and indirectly activates protein kinase C (PKC) *α *and *β *isoforms [26,31]. PKC*α/β *could then phosphorylate the protein p47phox [32,33]. Phosphorylated p47phox induces assembly of the cell membrane-associated six-protein complex that gains NADPH oxidase activity and therefore produces reactive oxygen species (ROS),[27,28,32,33] contributing to HIF-1 accumulation.

However, in this case the contribution from MAP kinase pathways is also essential. MAP kinase pathways driven by apoptosis signal-regulating kinase 1 (ASK1) are recruited. ASK1 and its downstream p38 MAP kinase and ERK (its activation does not depend on the ASK1) were found to contribute to the LPS-induced, TLR4-dependent accumulation of the HIF-1*α *protein [26,34]. LPS-dependent TLR4 signaling triggers cross-talk of ASK1 and HIF-1*α *protein [26]. ASK1 is selectively required for the activation of p38 MAP kinase during TLR4 downstream signaling [35,36]. p38 contributes to HIF-1*α *protein stabilization by its direct phosphorylation [26,37]. Earlier reports have demonstrated that ERK is also critical for TLR4-mediated HIF-1*α *accumulation [34].

Endosomal TLRs 7 and 8 trigger differential mechanisms to activate HIF-1*α *protein. In this case, a redox-dependent mechanism similar to the one observed for TLR4 takes place. However, in this case IRAKs 1 and 4 were also found to indirectly contribute to the activation of NADPH oxidase and therefore to influence ROS generation [25]. Earlier studies reported the direct contribution of IRAK1/4 to the phosphorylation of p47phox and causing the assembly of the NADPH oxidase complex during TLR signaling [37]. Reactive nitrogen species (RNS) are also involved in TLR7/8-induced activation of HIF-1*α *protein [25]. ASK1, PI3K, ERK, and p38 do not influence HIF-1*α *accumulation and HIF-1 transactivation in this case [25]. Although HIF-1*α *protein is one of the potential targets for posttranslational modification *via *its direct S-nitrosation [21,38]. This type of protein modification is not a major contributor to the TLR7/8-mediated HIF-1*α *accumulation [39]. ROS in the case of TLR4, ROS/RNS in the case of the TLR7/8, down-regulate HIF-1*α *prolyl hydroxylation leading to the accumulation of the protein, because of an inability to interact with the VHL protein [39]. NADPH oxidase-derived ROS were reported to negatively influence HIF-1*α *PHDs in other cases such as intermittent hypoxia [40]. Another report demonstrated a decrease in the expression of these enzymes in the cells exposed to LPS [24]. Our studies have demonstrated a decrease but not an attenuation of HIF-1*α *prolyl hydroxylation in human myeloid macrophages in response to LPS exposure [39].

**Figure 1:**
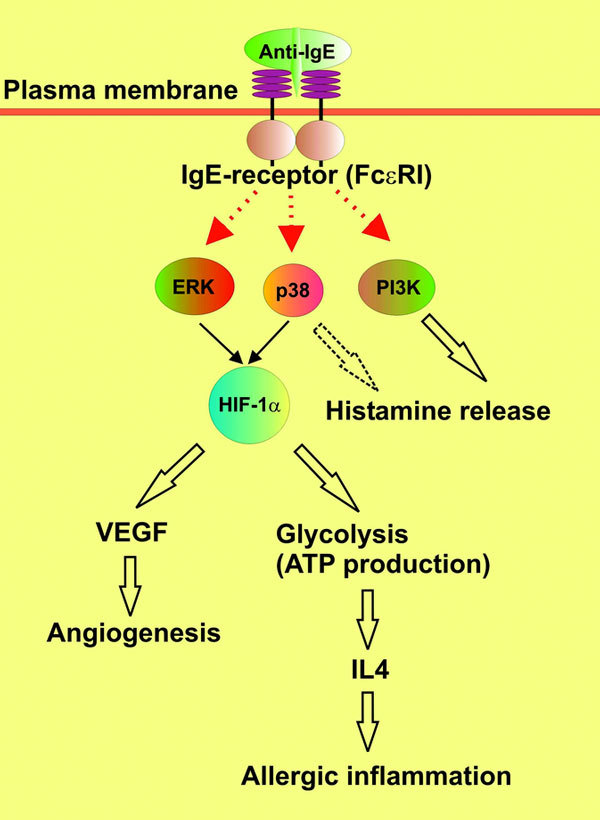
**Biochemical mechanisms of HIF-1*α *protein accumulation and its differential functional role in IgE-mediated responses of basophils**. Mechanisms leading to the accumulation and activation of HIF-1*α *protein during IgE-induced proallergic responses are presented. Differential functional roles of HIF-1 in cellular adaptation to inflammatory stress is summarized.

The HIF-1*α *protein is also known to be stabilized in basophils during allergic inflammation [18,40]. This HIF-1*α *accumulation has been found to occur in an IgE-dependent manner [18]. However, in this case, both ERK and p38 MAP kinase contribute to HIF-1*α *protein accumulation. Interestingly, ASK1, ROS, and PI3K did not influence anti-IgE-induced HIF-1*α *accumulation in primary human basophils [18]. The results showing changes in accumulation of HIF-1*α *protein upon pretreatment of basophils with pharmacological inhibitors of p38, ERK, and PI3 kinase before exposure to anti-IgE were inconsistent with those obtained with histamine release associated with degranulation. These experiments suggest that PI3 kinase is the major contributor to IgE-induced histamine release, which is also supported by the p38 MAP kinase. HIF-1*α *accumulation (not expression) was not dependent on PI3 kinase; however, it was affected in the case of attenuated ERK/p38 activities [18]. The mechanisms of the IgE-induced HIF-1*α *accumulation are summarized in the Figure 1.

## Differential functional role of the HIF-1 transcription complex in regulation of human inflammatory reactions

Functionally, the HIF-1 transcription complex is one of the major contributors to the inflammatory process. As the transcription factor triggered by hypoxia or stress, HIF-1 controls the adaptation of effector immune cells to inflammatory stress, protecting them against quick apoptosis and allowing them to generate an inflammatory response (eg, production of proinflammatory cytokines). In the cases of both infectious and allergic inflammation, HIF-1 supports energy metabolism, especially glycolysis, and, as such, prevents ATP depletion. In addition, HIF-1 contributes to the tracking of the ASK1 activity (in the cases when ASK1 activation takes place) preventing over-activation of the enzyme that would lead to quick and often irreversible activation of apoptotic signaling pathways inducing programmed cell death [18,25,41,42]. The HIF-1 transcription complex controls the expression of the protein phosphatase PP5 known to specifically down-regulate ASK1 activity [43]. By supporting cell survival, HIF-1 supports the production of proinflammatory cytokines (IL-6 and TNF-*α*) in innate immune cells during TLR-mediated inflammatory processes [25,42]. Importantly, recent evidence demonstrated the role of HIF-1 in expression of the cell membrane-associated TLR4 [44].

Expression of the proallergic cytokine IL-4 is also supported by HIF-1 in a similar way [18]. In human basophils, HIF-1*α *facilitates cellular adaptation to IgE-mediated stress and promotion of cytokine expression. However, these processes are differentially regulated compared with degranulation and histamine release [18].

In both kinds of inflammation the HIF-1 transcription complex directly controls expression of VEGF, thus regulating angiogenic processes [3,18,25]. Adhesion of immune cells and neovasculature also depends on HIF-1, which regulates the expression of CD18*β*2 adhesion integrins. Adhesion of immune cells is vital for the proper innate immune response and its correct linking to adaptive immune reactions. It is also crucial for the development of allergic reactions [4]. The functional role of the HIF-1 transcription complex in the allergic inflammatory responses is outlined in Figure 1.

Recent clinical studies have demonstrated that the HIF-1 transcription complex and VEGF play an important role in inflammation and hypervascularization observed in cases of bronchial asthma [45,46]. This suggests that further in vivo studies are required to investigate the concept described in this paper.

All these important functions are not yet a definitive list in terms of fully describing the contribution of the HIF-1 transcription complex in inflammatory processes and several aspects of HIF-1 activation during infections remain unclear. In the case of allergic inflammation, further complex studies have to be performed to clarify HIF-1 activation mechanisms in human basophils and mast cells and its functional role in pathologic cell reactions associated with allergic responses and disorders.
